# Conference Report: DATA ADMINISTRATION MANAGEMENT ASSOCIATION SYMPOSIUM Gaithersburg, MD May 11–12, 1993

**DOI:** 10.6028/jres.098.049

**Published:** 1993

**Authors:** Judith Newton

**Affiliations:** Information Systems Engineering, Computer Systems Laboratory, National Institute of Standards and Technology, Gaithersburg, MD 20899-0001

## 1. Introduction

Along with capital and human resources, an organization’s data represents one of its fundamental assets. Data administration (DA) attempts the effective planning, organization, and management of an enterprise’s data resource, with the intention of empowering the organization to achieve its mission and goals.

Reengineering the business processes, and the supporting information technology infrastructure, is a critical need for organizations. Yet despite substantial attention by business managers, technologists, and vendors of tools and methodologies, there are few obvious solutions or guidance on how to accomplish this.

The Data Administration Management Association (DAMA) is the professional organization for data administrators. An international board oversees a loose federation of local chapters in the United States, Canada, Australia, and Europe. The National Capital Region Chapter (NCR DAMA) has monthly meetings from September through April, as well as a Symposium in May.

NCR DAMA held its sixth annual Symposium at NIST on May 11–12, 1993. The theme this year was *Business Reengineering–The Competitive Edge.* Attended by over 200 Federal and private industry data administrators, the Symposium was cosponsored by NIST and NCR DAMA.

It emphasized the practices, technologies, activities, initiatives and ideas that deliver clearly visible value to the users, or “customers” of data administration. In addition to presentations by nationally recognized experts and practitioners, it included a workshop and panel discussions. Topics ranged from the keynote speech on reengineering business for the information age to the latest implementation of the Information Resource Dictionary System (IRDS) standard.

## 2. Speakers

The keynote speaker was Ron Shelby, of Connecticut Mutual Life Insurance Company. He has guided Connecticut Mutual through a complete redesign of its methods for handling insurance processing. The challenge of reengineering consists of moving away from the antiquated methods of mass production, assembly line, and cost-plus pricing to new assemble-to-order, flexible assembly, market-sensitive pricing practices. When a business can move from systems tracking things to systems driving events, results will be a change from high price and high quality to low price and high quality. Electronic information systems can be the tool to accomplish these business miracles. The steps needed are: first, analyze what is really needed; rearchitect the information; reengineer processes; retool with technology; and finally, reorchestrate and realign the workplace.

Cathy Hirsh of American Management Systems addressed the need to incorporate legacy data and processes in the design for reengineering. Organizations cannot afford to throw away and redevelop systems from scratch. Legacy data can be used as a leverage point to develop migration strategies incorporating the information warehouse concept as a stepping stone along the journey to true reengineering.

Larry English of Information Impact International discussed the need for good data management and how it can support business re-engineering. Data modeling provides stability and flexibility for the process. Data management can enable the paradigm shift in business areas by establishing business accountability for data and a single source of data capture. Single data maintenance applications by data type and life cycle must be in effect. Processes to support reengineering include focussing on strategic uses of data, establishing metrics, and keeping lines of communication open.

As an organization strives to improve its operations, the business leaders must determine the value of each process in terms that are meaningful to the operation. Ron Gustafson of EDS defined a value construct by which each business process may be evaluated before any automated system is designed, built, or selected to support that process. As the business leaders reengineer their organization’s processes, they can establish, through this value construct, the set of fully integrated business processes needed to deliver optimal results to the enterprise. He illustrated how the value construct would be applied to a hypothetical business and discussed the potential benefits to both the business organization and to the information technology service provider.

Ron Ross of Ronald G. Ross Associates discussed modeling for enterprise-level reengineering. Resources include a set of subject-level databases, far more flexible than traditional application-area databases and therefore more suitable for reengineered processes. A tool to establish these subject-area databases, resource life-cycle analysis, determines the value chain and precedence of processes within each resource chain.

Mike Yoemans and Bunny Smith of DoD presented an overview of the Corporate Information Management (CIM) effort to integrate information systems within the Department of Defense. The process involves department-wide built business processes, shared data structures, a business modernization focus, matrix management organization, functional management of organization, architecturally driven philosophy, source data entry, common work breakdown structure, single code of accounts, data administration, life cycle program and project measures, and a standard hardware/software/communications platform.

The last speaker, John Zachman of Zachman International, presented an explication of his Information Architecture concept together with his personal view of the future of manufacturing and technology in the near future. We must change from Make-to-order and Provide-from-stock to Assemble-to-order processes. He issued a challenge for everyone in the audience to rise to meet the challenge of “The New Realities of the Information Age.”

## 3. Panels

The following panel discussions were presented:

- Modeling Languages, Sandra K. Perez, Concept Technologies, moderator;
- DAMA/NCR Government Special Interest Group, Tom Kurihara, NIST, and Peter Aiken, DoD, moderators;
- Standards for Reengineering, Judith J. Newton, NIST, moderator;
- Functional Economic Analysis, Carla von Bernewitz, Vector Research, moderator;
- Data Administration Support for Business Process Improvement, Duane Hufford, AMS, moderator;
- Tangible Benefits of Reengineering, Cindy Walker, Software Solutions, moderator;
- Information Architecture, Raghu Chintala, Booz, Allen and Hamilton, moderator.

## 4. Standards and Procedures Workshop

The Working Group on Standards and Procedures has been meeting monthly during the past year. It has produced the Manual for Data Administration, NIST Special Publication 500-208. A copy of this publication was supplied to each Symposium attendee at registration. Additional copies are available from the author of this Conference Report. This document discusses a wide range of data administration topics, including development and implementation of standards.

The Standards and Procedures workshop at this Symposium presented the draft result of the group’s current effort: development of a survey to be distributed to data administrators in an effort to determine the current state of development of the art.

## 5. Proceedings

The proceedings of this, the Third, and the Fifth Symposia were distributed at the event. The Proceedings of the First,^1^ Second,^2^ and Fourth^3^ Symposia were published by NIST and copies are still available. The Seventh Annual Symposium will be held May 17–18, 1994.

## Footnotes

1 Judith J. Newton, and Frankie E. Spiclman, eds., *Data Administration: Management and Practice, Proceedings of the First DAMA Symposium*, NIST Special Publication 500-159, National Institute of Standards and Technology, Gaithersburg, MD, October, 1988.

2 Judith J. Newton, and Frankie E. Spiclman, eds., *Data Administration: Standards and Techniques, Proceedings of the Second Annual DAMA Symposium*, NISTIR 90-4292, National Institute of Standards and Technology, Gaithersburg, MD, April 1990.

3 Judith J. Newton, Mary Lou Melley, and Holly Higgins, eds., *Managing Data: From Vision to Reality, Proceedings of the Fourth Annual DAMA Symposium*, NISTIR 4843, National Institute of Standards and Technology, Gaithersburg, MD, May 1992.

